# The raters’ differences in Arabic writing rubrics through the Many-Facet Rasch measurement model

**DOI:** 10.3389/fpsyg.2022.988272

**Published:** 2022-12-16

**Authors:** Harun Baharudin, Zunita Mohamad Maskor, Mohd Effendi Ewan Mohd Matore

**Affiliations:** ^1^Faculty of Education, Center of Diversity and Education, Universiti Kebangsaan Malaysia (UKM), Selangor, Malaysia; ^2^SMK Khir Johari, Tanjong Malim, Perak, Malaysia; ^3^Faculty of Education, Research Centre of Education Leadership and Policy, Universiti Kebangsaan Malaysia (UKM), Selangor, Malaysia

**Keywords:** raters, analytic rubric, writing assessment, Many-Facet Rasch measurement model (MFRM), writing domains, Arabic essays validation

## Abstract

Writing assessment relies closely on scoring the excellence of a subject’s thoughts. This creates a faceted measurement structure regarding rubrics, tasks, and raters. Nevertheless, most studies did not consider the differences among raters systematically. This study examines the raters’ differences in association with the reliability and validity of writing rubrics using the Many-Facet Rasch measurement model (MFRM) to model these differences. A set of standards for evaluating the quality of rating based on writing assessment was examined. Rating quality was tested within four writing domains from an analytic rubric using a scale of one to three. The writing domains explored were vocabulary, grammar, language, use, and organization; whereas the data were obtained from 15 Arabic essays gathered from religious secondary school students under the supervision of the Malaysia Ministry of Education. Five raters in the field of practice were selected to evaluate all the essays. As a result, (a) raters range considerably on the lenient-severity dimension, so rater variations ought to be modeled; (b) the combination of findings between raters avoids the doubt of scores, thereby reducing the measurement error which could lower the criterion validity with the external variable; and (c) MFRM adjustments effectively increased the correlations of the scores obtained from partial and full data. Predominant findings revealed that rating quality varies across analytic rubric domains. This also depicts that MFRM is an effective way to model rater differences and evaluate the validity and reliability of writing rubrics.

## Introduction

Writing skills are complex processes and require the coordination of various high metacognitive skills. In order to produce written ideas, a writer must be able to organize and generate ideas, develop plans for ideas, review the writing, and monitor self-esteem in writing (Olinghouse and Leaird, 2009; Dunsmuir et al., 2015). Writing skills must be mastered in foreign language learning as stated explicitly in the curriculum document. Writing is the ability of a human to communicate in a set of letters that become understandable sentences. Through writing, students’ thinking might be highlighted through the way it is organized, combined, developed, and strived to create an association of ideas to assist readers to understand their thinking organization. Based on writing performance in writing on gender, Adams and Simmons (2018) emphasized that there were significant gender differences in the writing performance of year 1 and year 2 among children from the North West of England, where boys produced shorter essays with fewer words spelled correctly, and were rated lower than girls. Findings also concluded that female students are more capable of producing quality writing than male students.

Writing skills are productive and expressive, and both are important as information conveyers. Writing skills are productive because writing is a productive activity of written work in the form of the expression of one’s thoughts. Whereas expressive implies appropriate (able to) give (expression) images, intentions, ideas, and feelings (Mufidah et al., 2019). Writing skills are skills that are difficult for students to teach and master (Kurniaman et al., 2018) because it involves the most complex level of literacy and requires a high level of cognition. Mastery of writing skills can make a person more confident in speaking because aspects of vocabulary, grammar, and sentence structure have been mastered well.

The production of a piece of writing is the highest level in writing skills, which is the skill of producing an essay have harmony of words and meaning. The basic skills in writing are constantly developing and becoming more complex, especially when it comes to high-level writing skills that involve more complex knowledge about oral language skills, including knowledge of vocabulary and word retrieval as well as grammar and syntax. High-level writing also requires proficiency in the use of executive functions such as planning and working memory involved in the generation and transformation of ideas into words (Decker et al., 2016).

Refers to the context of writing skills in learning Arabic; it is a language skill that is emphasized a lot. This is in line with the content of textbooks that are more inclined to a written assessment format than listening, speaking, and reading (Mahmood and Zailaini, 2017). Students also need to be good at writing because most of the exam papers in Malaysia require answers in written form. Students who do not master writing skills often have difficulty answering questions to convey information and answers accurately.

Conducting a study to assess the mastery of writing skills is a necessity. The aspect of vocabulary knowledge is one of the aspects of language that need to be paid attention to in writing skills in addition to aspects of grammar, organization of ideas, and language style. In fact, vocabulary is also an aspect of the language that needs to be mastered, as stated in the Arabic curriculum document. There are several issues regarding the assessment of Arabic language writing. The first issue is that Arabic language teachers usually only focus on teaching grammar to the point of marginalizing the importance of vocabulary mastery (Maskor et al., 2016). While foreign language learning at this point focuses more on the mastery and use of words in the target language as implemented in the Common European Framework of References for Languages (CEFR) as suggested by Alderson (2005).

In line with this requirement, this research aims to examine written assessment in Arabic using an expository essay, which provides valid, accurate, and fair ratings that are compatible with students’ word acquisition in Form Four for religious schools in Malaysia. There are four aspects of language that are emphasized in the assessment of writing skills based on the rubric of the Malaysian Certificate of Education (SPM) Arabic Trial Exam in the State of Selangor, namely, (1) vocabulary, (2) grammar, (3) language style, and (4) aspects of text organization before formulated into the overall test score for students’ writing skills.

## Literature review

The designed writing assessments are a reflection of language learning purposes. The essay produced by a student is an indicator of the student’s ability to master a foreign language communicatively (Bachman and Palmer, 1996). In line with the theory requirements, Bachman and Palmer (1996) suggested that the assessment is aimed at a real-life simulation that includes individual performance and performance appraisal by raters. Although McNamara (1996) argued that the communicative theory is still relevant in testing language ability; hence, different aspects need to be considered before setting the level of writing abilities and scoring processes at school levels.

Examining the facts in-depth, Phelps-Gunn and Phelps-Terasaki (1982) and Khuwaileh and Shoumali (2000) identified three aspects of students’ weaknesses in writing: (a) a description that indicates the sentences are not elaborative, non-specific, and characterized by simple word usage, slang usage, and incomplete ideas; (b) writing styles that are less clear, not focused on topics, less integrated, less logical, less emphasis, and less consistent with writing goals; and (c) error in the punctuation, grammar, mechanical, spelling, and capitalization. Based on this weakness analysis, a scoring scheme for writing skills is triggered.

In line with Bachman and Palmer (1996), the evaluation process is a strategic competency that includes the description of the assigned tasks and the scoring rubric. Scoring rubrics are methods of controlling the reliability and validity of student writing results. Several researchers noted the evaluation of educators is more accurate when using the rubric (Jonsson and Svingby, 2007; Rezaei and Lovorn, 2010). Meanwhile, the adverse impact of using rubrics, such as the low reliability has yet to be elucidated in several studies. Consequently, many educators employed rubrics with the premise that they improve grading objectivity, especially regarding the written submissions of learners. Several empirical studies have raised serious doubts about the validity of rubric-based performance assessments, such as Sims et al. (2020) and Mohd Noh and Mohd Matore (2022).

Weigle (2011) categorizes three types of writing rubrics, namely, analytical, holistic, and primary trait. Primary trait rubrics are mostly used to determine learners’ necessary writing abilities concerning particular writing tasks. Holistic rubrics are used to evaluate the characteristics of learners’ written works by utilizing an in-line score with the determined characteristics and superficially described distinct performance levels such as grammar, spelling, and punctuation mistakes (Gunning, 1998; Weigle, 2013).

Holistic scoring is difficult for second-language learners as distinct elements of writing skills evolve differently for various writers. Wiseman (2012) concluded that some students might express strong content and organization but are limited in grammatical precision, while others can exercise excellent sentence language control but are unable to organize their writing. Some students may not perform similarly, for every component of written ability, necessitating more quality assessment methods such as lexical, syntactic, speech, and rhetorical characteristics.

A study conducted by Knoch (2011) comparing holistic rubrics with analytics revealed that the rater reliability was significantly higher and raters could better differentiate between various aspects of writing when more detailed analytical scale descriptors were used. Hence, analytic rubrics are more comprehensive evaluation guides used to clarify the level of expertise in distinct areas of written tasks (Vaezi and Rezaei, 2018). In addition, Winke and Lim (2015) clarified that raters attended all the scoring categories described in the rubric, while concentrating on what they felt was essential with the holistic rubric.

Earlier studies have shown that raters have a significant effect on written assessment results. Researchers recognize the mediating importance of rater judgment in student writing (Eckes, 2008; Engelhard, 2013; Zhang, 2016). In other words, many researchers are interested in the degree to which rating errors and systemic biases introduce irrelevant structural variation in the interpretation of ratings. Concerning rater impacts, features like rubrics can also lead to psychometric constraints in rater-mediated writing assessments (Hodges et al., 2019).

The present study emphasizes that raters have several variables to address when rating and participating in tasks that require an assessment from various information sources. However, in contrast to the studies conducted in the Arabic language, written assessment receives less attention and emphasis on empirical validation and reliability. Therefore, it is essential to monitor rater quality in terms of their usage of rating scales and contribution to test the validity and reliability. The current study focuses on the examinees (essays), raters, writing domain (rubric), and rating scale for 15 Arabic essays. Thus, the analytical method was selected in order to control the consistency of raters’ ratings based on the designated scoring criteria.

This research aims to examine written assessment in Arabic using an expository essay, which provides valid, accurate, and fair ratings. Concurrently, this study provides information on the characteristics of effective writing. We used a scoring rubric from previous examinations that inform raters about high-quality writing throughout the scoring process. These rubrics also minimize discrepancies between raters given the distinct instrument interpretations. The following sections present the sample, rating processes, and data analysis procedures that demonstrate the validity, reliability, and fairness of the data scores. Specifically, the following research questions are to be answered in this study:

a. To what extent do the interpretation and use of writing domains in the rubric demonstrate validity?
b. To what extent do the interpretation and use of writing domains in the rubric demonstrate reliability?
c. To what extent do interpretation and use of writing domain in the rubric demonstrate fairness?

## Materials and methods

This study uses an analytic scoring rubric adopted from previous state examinations for the Malaysian Certificate of Education Trial Examination for Arabic. The main reasons of doing this research topic is because it has highly needs to examine written assessment in Arabic which will provides valid, accurate, and fair ratings. Previously, not much was discussed regarding empirical evidence about the accuracy of assessors in giving accurate assessments related to Arabic writing. Formerly, evaluation was limited to the use of analysis such as Cohen Kappa, which is more limited to raters. The main instrument/examinee in this study are 15 essay selections based on the writing performances of the respondents. The essay was produced by Form Four students from a religious secondary school, which is under the supervision of the Ministry of Education. However, the topic of the proposed essay is one of the themes in the Arabic language syllabus, which is written in the textbooks. Therefore, it is not a peculiar matter for respondents to write an essay according to the selected title. The essay selection was based on a researcher’s brief assessment of the essay quality ranging from good to moderate and weak. The essay was collected and printed in one booklet. Rubric scoring guides and rating scales were provided for each essay. The layout print was used to enable the raters to review the essays.

Five proposal titles were chosen for expository essays to gain experts’ agreement. First, the procedure for determining the content validity of the writing task involves five experts in the selection of appropriate essay titles and scoring rubric items. At this phase, the content validity index (CVI) was applied to determine the expert agreement scores. The CVI covers the validity of the item (I-CVI) and that of the entire instrument (S-CVI) (Lynn, 1986). Each expert evaluates the level of item suitability based on four-level scales, where 1 = very inappropriate, 2 = inappropriate, 3 = suitable, and 4 = very suitable. In the first round, each expert is given at least 2 weeks to confirm the items proposed by the researcher and is asked to suggest improvements to the item if any. After 2 weeks, the researcher re-contacted the expert for the confirmation of the proposed item. The second round was conducted after the researcher made an improvement or correction based on the proposals received from the experts.

The value of I-CVI was used to determine the reliability between the experts in line with the average level of suitability of each item based on the assessment of all appointed experts. The accepted I-CVI value is 1.00 based on the value of the five expert’s agreement. Meanwhile, the S-CVI value of the essay is 0.91. Polit and Beck (2006) suggested that an S-CVI value of >0.80 is an indication of the overall acceptable quality of the item. The higher the value of S-CVI, the higher the quality of the item and the choice of the expert in meeting the criteria of the instrument. These five experts also served as the raters for examining the internal consistency of rubrics for essays that were evaluated using the MFRM.

Students were informed that the essay was to assess students’ knowledge of vocabulary and their ability to construct context-related sentences. Students were asked to write no fewer than 40 words based on their creativity within 30 min. The five raters were then given the five essays to evaluate. Five raters gave each essay a score, and the amount of connectivity needed for a Rasch analysis was found. The five raters were asked to rate the essays using the Malaysian Certificate of Education Trial Examination for the Arabic rating scale (analytical rating). The rubric is analytical, which comprises domains of writing known as vocabulary, grammar, language use, and Organization as agreed by experts. Every domain contains three scales: excellent (score 3), moderate (score 2), and weak (score 1). The reasons for choosing the analytical rubric are because it can explicitly segregate an assignment into its constitutive skills and provides the assessor with guidelines for what each performance level looks like for each skill.

The selected scoring rubric is from a previous state examination for the Malaysian Certificate of Education Trial Examination for Arabic. This examination was implemented in one of the states in Malaysia in 2015. The use of a scoring rubric is based on assumptions that the scoring format is similar to the summative test, which is commonly used for school-based assessment. Although the scoring rubric has been used widely, its validity and reliability have to be tested according to the scope of this study. In contrast, the respondents of this study are also exposed to such essays in the classroom.

The essay selection is the result of the respondents’ writing performances. They were 15 essays of Form Four students from a religious secondary school, which is under the supervision of the Ministry of Education. However, the topic of the proposed essay is one of the themes in the Arabic language syllabus, which is written in the textbooks. Therefore, it is not a peculiar matter for respondents to write an essay according to the selected title. The essay selection was based on a researcher’s brief assessment of the essay quality ranging from good to moderate and weak. The essay was collected and printed in one booklet. Rubric scoring guides and rating scales were provided for each essay. The layout print was used to enable the raters to review the essays.

Meanwhile, the selection of raters was based on their experience in Arabic language education. Two teachers are expert teachers in Arabic who have been teaching for more than 10 years in secondary schools. In contrast, two more raters are experienced teachers who have been teaching Arabic for over 20 years in a religious secondary school that is under the supervision of the Ministry of Education. Another teacher is a novice teacher who has 5 years of teaching experience in Arabic in a secondary school. They were chosen because of the research to evaluate the rubrics used as well as validate them using the Malaysian Certificate of Education Trial Examination for Arabic rating scale. Table 1 summarizes the criterion of each rater.

**TABLE 1:** Summary of rater criteria.

Rater code	Workplace	Subject	Working experience
A	Secondary school	Arabic Language Upper Form	18 years
B	Secondary school	Arabic Language Lower Form	15 years
C	Religious secondary school	Arabic Language Lower Form	22 years
D	Religious secondary school	Arabic Language Upper Form	25 years
E	Secondary school	Arabic Language Lower Form	4 years

This study employed the Many-Facet Rasch measurement model (MFRM) model to explain how the rater interpreted the scores of the writing tasks. For this research context, the Rasch model is extended by MFRM to situations in which more than two facets interact to produce an observation in Arabic writing. It enables the development of a frame of reference in which quantitative comparisons are no longer dependent on which examinee was rated by which judge on which item. In order to support the research in the Arabic language, this analysis seeks to evaluate the rubrics used as well as validate them. Raters mediate the scores of the essays. In other words, self-rating does not represent the writing quality of the test directly as the rater’s judgment plays a crucial role (Engelhard and Wind, 2017; Jones and Bergin, 2019). Hence, research is needed to review scores on quality writing and the consequences of scoring tasks.

A previous study used MFRM to investigate the variations in rater’s severity and consistency before and after practice and found that rater training contributed to increasing the accuracy of the scorer’s intra-rater reliability (internal consistency) (Weigle, 1998). Lim (2011) also conducted a longitudinal study for 12–21 months among novice and skilled raters to examine rater harshness/leniency, accuracy/inactiveness, and centrality/extremism. The study of the different impacts of the written rater can lead to better scores and rater training. It can also provide validation data on rating scales of writing assessment (Shaw and Weir, 2007; Knoch, 2011; Behizadeh and Engelhard, 2014; Hodges et al., 2019).

The findings could portray in-depth that the MFRM can monitor rater’s performances and considers the potential effects of facets on the resulting scores. Facets such as raters, rating scales, and examinations are arranged within the standard interval scale with rater scores (Goodwin, 2016; Eckes, 2019). Two assumptions were made to draw meaningful information from MFRM measures: The data must fit in with the model, and the test must measure a single unidimensional construct.

The raw data were keyed-in using Microsoft Excel and analyzed using the MFRM by the FACETS 3.71.4. A program named FACETS 3.71.4 was used to analyze the data in MFRM. This study represents the relationship between facets assessment and the probability in which specific results will be observed within more than one-faceted circumstances. In addition, this research is an expansion of the Rasch measurement theory (Engelhard and Wind, 2013), in which raw scores are transformed into log odds. This interval scale implies that an equivalent range between any two information points is equivalent to the capacity of individuals or items (Bond and Fox, 2015). The FACETS program can produce the interval scale as a variable map or Wright map for direct comparisons of the test-taker writing proficiency, raters’ severity, scale difficulty, or other facets of interest (Eckes, 2019). Briefly, there were three facets in this research: raters, examinee (expository essays), and scoring rubric or item (analytical rating elements: vocabulary, grammar, language use, and organization).

This variation in the MFRM enables a classification scale framework to differ by item in this situation. The MFRM can demonstrate discrepancies among raters in the use of scoring classifications (Engelhard and Wind, 2013). The Many-Facet Rasch measurement model used in this analysis can be expressed as:


(1) $${\text{P}}_{\text{nikj}}=\frac{{\text{e}}^{\left(\beta_{\text{n}}-\delta_{\text{i}}-{\text{F}}_{\text{k}}-{\text{C}}_{\text{j}}\right)}}{1+{\text{e}}^{\left(\beta_{\text{n}}-\delta_{\text{i}}-{\text{F}}_{\text{k}}-{\text{C}}_{\text{j}}\right)}}$$

where β_n_ represents individual ability, δ_i_ represents the level of scales difficulty, F_k_ represents the level of threshold difficulty, and C_j_ represents the level of rater’s efficiency.

## Results and discussion

Table 2 shows the essay, rating scale, and raters’ reliability index based on the MFRM approaches using Facet 3.71.4 software. The findings indicate that the mean logit of the essay is at 0.00 logit with a standard deviation (SD) of 1.29. This finding reflects broad dispersion throughout the logit scale. This widespread ability level denotes the presence of various levels of essay quality. The rating scale at SD = 0.32 illustrates that the dispersions are not so vast on the logit scale, and this finding is equivalent to the raters at SD = 0.46. However, the average MNSQ outfit for the essay is 0.97, as the rating scale (0.97) and the raters (0.98) are approaching the expected value of 1.00. Therefore, based on the SD values for the essay, the rating scale and raters establish that the instrument aligns with the model. The chi-square values for raters (15.5) and essays (105.1) are significant Engelhard (2013), whereas the rating scale does not reveal a significant value. Further analysis needs to be conducted to ensure the rating scale is reliable.

**TABLE 2:** Summary statistics of essays, rating scale, and raters’ reliability.

	Essays	Writing domains (Rubric base rating scale)	Raters
N	15	4	5
Measures			
Mean	0.00	0.00	0.01
Standard deviation (SD)	1.29	0.32	0.46
Standard error (SE)	0.45	0.23	0.26
Outfit mean-square			
Mean	0.97	0.97	0.98
Standard deviation (SD)	0.31	0.17	0.18
Homogeneity index (x2)	105.1*	7.6	15.5*
Degree of freedom (Df)	(15−1) = 14	(4−1) = 3	(5–1) = 4
Strata	3.88	1.59	2.27
Reliability	0.88	0.47	0.68
Inter-rater reliability			
Observed exact agreements			52.9%
Expected %			55.0%

*p < 0.01.

The reliability of the essay is 0.88, while the separation index is 3.88, thus indicating good reliability (Fisher, 2007). Whereas the reliability of the raters is 0.68, and the separation index is 2.27, thereby corresponding to moderate reliability and acceptable separation (Linacre, 2006; Fisher, 2007). The rating scale (0.47) demonstrates poor reliability (Fisher, 2007), but the separation index (1.59), which equals two separation indices, denotes a good item separation (Linacre, 2006). Although statistical findings revealed a non-homogeneous rubric with low-reliability values and non-significant chi-square, the separation index illustrates that the raters understood the rubric base rating scale.

Raters displayed reasonable agreement based on the value of the inter-rater reliability of 52.9%, which was not different from the 55.0% despite the moderate reliability. These findings may elaborate that the raters had the same opinion in scaling the essay rating and vice versa (Linacre, 2004). Overall, the reliability value for essays, rating scales, and raters is reasonable and acceptable. Validation analysis in MFRM includes fit statistics and scale calibration analysis. Fit statistics is one of the validation indicators by observing the mean square, Z-standard (Z-std), and point-measure correlation values. Table 3 shows essay number 11 to be out of range in terms of mean square (0.5–1.5) (Linacre, 2005; Boone et al., 2014), thereby exhibiting an adverse polarity. Whereas essay numbers 7, 8, and 11 demonstrate negative polarities, which denote that the content does not fit the topic. Essay numbers 2, 9, 10, 13, and 14 are less than 0.30 point-measure correlation values, indicating that the essays are unable to highlight the respondents’ abilities (Linacre, 1999; Bond and Fox, 2015). Essay number 15 is considered the weakest at logit (−3.40), disclosing many errors but still on the topic. Meanwhile, essay number 12 is the best essay as it occupies the highest logit position (1.76). This finding indicates that some participants are unable to effectively compose essays even if the topic of selection is a prevalent subject in the formative and summative tests.

**TABLE 3:** Analysis of fit statistic for essay.

Essay number	Measure	Model S.E	Outfit		PTMEA CORR
			MNSQ	Z-std.	
1	0.41	0.45	0.61	–1.1	0.46
2	0.41	0.45	1.19	0.6	0.13
3	1.57	0.44	1.19	0.8	0.78
4	1.57	0.44	1.08	0.4	0.44
5	–0.19	0.45	1.16	0.5	0.52
6	–1.55	0.44	0.72	–1.2	0.48
7	–0.79	0.44	0.84	–0.4	–0.27
8	0.80	0.44	0.76	–0.7	–0.09
9	–0.59	0.45	0.87	–0.2	0.22
10	–0.39	0.45	1.18	0.6	0.16
11	–0.39	0.45	1.69	1.7	–0.02
12	1.76	0.44	1.20	0.9	0.30
13	0.01	0.45	0.40	–2.0	0.22
14	–0.80	0.44	1.00	0.1	0.13
15	–3.40	0.56	0.74	–0.5	0.43

Table 4 shows the appropriate rubric base rating scale statistics in the MNSQ range from 0.50 to 1.50 (Linacre, 2005; Boone et al., 2014). The Z-std value was also within the range of +2.0 (Linacre, 2005; Bond and Fox, 2015) and PTMEA’s, which represents a value greater than 0.30 (Bond and Fox, 2015). These values indicate the item measures a single construct (Bond and Fox, 2015). Of the four proposed domains, the vocabulary element is readily understood by the logit raters (−0.41), whereas the grammatical elements are the most challenging (0.39). However, the standard error is an excellent range, which is stated by a value of <0.25 (Fisher, 2007). Overall, the rating scale disclosed that all rating scale elements are fit and suitable for the evaluation and measurement of the essays to be performed. All raters also deeply comprehend the rating scale.

**TABLE 4:** Analysis of fit statistic for rubric base rating scale.

Writing domain (Rating scale)	Measure	Model S.E	Outfit		PTMEA CORR
			MNSQ	Z-std.	
Vocabulary	–0.41	0.23	1.09	0.5	0.71
Grammar	0.39	0.23	0.86	–0.7	0.53
Language use	0.23	0.23	1.18	1.0	0.50
Organization	–0.20	0.23	0.76	–1.4	0.51

Table 5 shows the statistical coefficients of five raters from codes A, B, C, D, and E, ranging from 0.50 to 1.50 (Linacre, 2005; Boone et al., 2014). The Z-std values are also within the range of +2.0 (Linacre, 2005; Bond and Fox, 2015). The overall value of PTMEA is 0.30, indicating that the raters can distinguish between each rubric used in the rating scale (Bond and Fox, 2015). Concerning the logit, rater B (logit 0.62) is the most stringent rater, while rater E (logit −0.78) is the most-lenient rater. The standard error value is quite good, within the range of <0.50 (Fisher, 2007). This value indicates that the rater evaluates the essay carefully. The results also reveal that they can use the rubric precisely based on their knowledge.

**TABLE 5:** Analysis of fit statistics for raters.

Rater	Measure	Model S.E	Outfit		PTMEA CORR
			MNSQ	Z-std.	
Rater A	0.01	0.26	0.92	–0.3	0.55
Rater B	0.62	0.26	0.74	–1.3	0.48
Rater C	0.24	0.27	1.07	0.4	0.67
Rater D	–0.06	0.25	0.97	–0.1	0.57
Rater E	–0.78	0.26	1.18	1.0	0.56

### Rating scale functioning

This calibration was analyzed using a rubric-based rating scale, where scale 3 = distinction, scale 2 = medium, and scale 1 = weak. In general, the variation in each rubric scale is in the appropriate range of 1.4–5.00 (Linacre, 1999, 2004), as shown in Table 6.

**TABLE 6:** Rating scale calibration.

	Data		Quality control			Rasch andrich thresholds		Expectation
				
Score	Category total	Counts used	Average measure	Expected measure	Outfit MNSQ	Measure	S.E	Measure category
1	54	54	–1.36	–1.51	1.1			(−2.96)
2	191	191	0.01	0.10	0.90	–1.88	0.18	0.00
3	55	55	1.30	1.12	0.90	1.88	0.17	(2.97)

**FIGURE 1:**
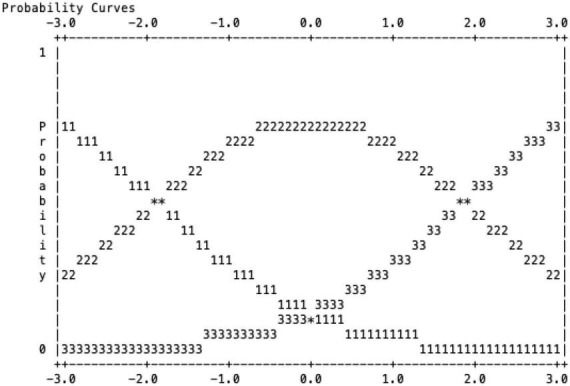
Probability curve of the rating scale.

Figure 1 also portrays a non-threshold scale of scale 1 with scale 2 at −1.88 and scale 3 at 1.88. This finding depicts that the scale curve is apparent and separated from each other (Figure 1). Figure 1 also defines that, in the assessment of the essay, each rater understands the function of each rubric. The scale ranking results in this study can be used for further research.

### Variable map (Wright map)

On average, the examinee (essays) locations were close to logit scales at the rater scale and rubric base rating scale (all-around zero logits). This measure suggests acceptable targeting between the three facets. Figure 1 provides additional information about the logit scale. Specifically, Figure 1 is a variable map that graphically displays the test-takers, raters, and the category of rating thresholds.

The first column indicates the estimated location of the logit scale of the test-takers (essay), raters, and item (rating scale). Higher numbers denote higher judged writing performance, more severe raters, and more difficult rating scale categories. The second column depicts the locations for essays. The examination of the essay locations reveals a wide range of locations between −3.40 and 1.76 logits for the lowest (mean rating = 1.18) and highest judged writing performances (mean rating = 2.45), respectively. The third column shows the locations for the item or writing domain for the rubric. The examination of these estimations reveals a range of item difficulties, between −0.41 logits for the item that was judged as easiest (item vocabulary; mean rating = 2.11) and 0.39 logits for the item that was judged as the most difficult (item grammar; mean rating = 1.91). The fourth column depicts the locations of the individual raters. The location estimates reflect differences in rater severity, ranging from −0.78 logits for the most-lenient rater (rater E; mean rating = 2.13) to 0.01 logits for the most severe rater (rater A; mean rating = 2.00). The final fifth column in the variable map illustrates the categories of the calibration of the rating scale ranging from scale 1 to 3.

The accuracy of the location estimation was assessed using SEs and separation statistics. Table 2 shows a small range of SEs for essays (0.45), raters (0.26), and rating scales (0.23) regarding the distribution of the logit scale. In particular, the average SE for the essay facet was relatively higher for the rater and the rubric base rating scale than the average SEs. This result is expected given the higher number of observations among each rater and every item in the rating scale compared with each student.

**FIGURE 2:**
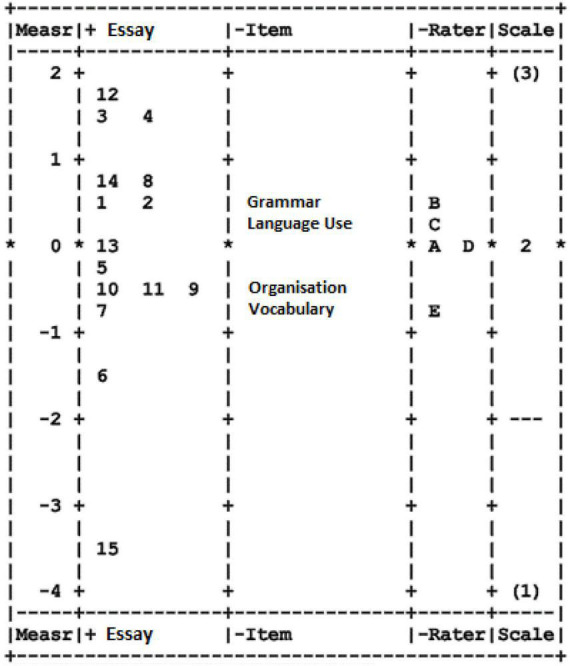
All facet vertical unit.

Figure 2 shows the descriptive mapping for each facet evaluated in this study. The first column is an essay using the value of “logit” (1.76 to −3.40), which describes the comparative quality of the essays (column Essay) tested. Essay number 12 is the most outstanding (1.76), while essay number 15 is the weakest because it is at logit (−3.40). Essays numbers 3, 4, 8, 12, and 14 are excellent category essays that conform to the writing scoring criteria. Essay number 13 fails in the aspects of grammar and language use. Essay number 5 only passes the vocabulary aspect, while essays numbers 7, 6, and 15 fail in all aspects of the rubric. Essay numbers 10, 11, and 9 fail to address all elements of grammar, language use, and organization but are likely to fail or pass in vocabulary.

The five raters who assessed the essay can be classified into three categories: stringent, moderate, and lenient. This measure can be seen in the fourth column (column Rater) in the range of −1 to +1. Rater E is considered the most lenient in scoring, while raters A and D are modest in scoring. Meanwhile, raters B and C are stringent raters in this essay scoring, and both of them taught Arabic in a lower form. Rater E is a novice teacher who only has 4 years of experience in teaching Arabic at a lower form in a secondary school. Rater B is an Arabic teacher who has taught Arabic for 15 years for lower form, and also rater C who taught Arabic for 22 years at a religious secondary school.

Rater E is a novice teacher who has taught the Arabic language for 4 years in a lower form at secondary schools. Meanwhile, raters A and B have been teaching Arabic at secondary schools for more than 10 years for upper and lower forms. In addition, rater C taught Arabic in the lower form, while rater D taught the upper form for more than 20 years at religious secondary schools. The diversity of the rater’s backgrounds reflects their performance in assessing the essay. The Arabic teachers who taught upper forms are considered a better scoring performance than those who taught lower forms. The performance displayed by raters A and D is likely to result from their experience in teaching senior high-school students. Notably, the type of school does not influence the rater’s performance. Conclusively, the variable map (see Figure 2) depicts that the rubric used for the rating scale can differentiate the quality of the essay produced by the respondent.

## Limitations and future research

Additional proof is required in various contexts to determine the psychometric features of the rubrics in writing. Specifically, a future study could investigate whether the new rubrics may be added to determine the efficacy and efficiency of each rubric rating in terms of reliability, validity, and fairness. The raters in this study demonstrated different seriousness levels when using the rubrics to evaluate the students’ outcomes. Statistically, the Rasch model alleviates rater severity in the calculation of test-takers’ results (Wright and Linacre, 1989). However, further research should provide significant reasons why raters use these rubrics for writing assessments to the extent of differences in seriousness. For instance, researchers could perform interviews with raters concerning their judgment procedures to understand how raters interpret and apply the rubric to student compositions. A future study among the population is required to determine whether the rubrics are fair among groups and individuals from different types of schools. Nevertheless, validity and reliability could be enhanced by involving more raters and essays in future studies.

## Conclusion

The findings from this study demonstrate strong validity, reliability, and fairness of scores. Overall, the Many-Facet Rasch measurement model (MFRM), which is rarely used in Arabic studies, reflected that the rubric for rating scores has good reliability and validity and can be used in actual studies. All raters can effectively differentiate the functions of each rubric and rating scale. The use of rubrics in scoring can detect the strengths and weaknesses of students in writing skills (such as language use, organization, grammar, and vocabulary use). The feedback from scoring could assist teachers in developing ideas regarding teaching strategies based on students’ weaknesses. The choice of the analytical method is more accurate than the holistic method in order to assess the writing performance. Moreover, an analytical method could be provided through the information on the mastery stage of each writing domain.

## Data availability statement

The original contributions presented in this study are included in the article/supplementary material, further inquiries can be directed to the corresponding author.

## Ethics statement

The studies involving human participants were reviewed and approved by Ministry of Education Malaysia, Putrajaya. The ethics committee waived the requirement of written informed consent for participation.

## Author contributions

HB was involved in data collection. ZM wrote the first draft of the manuscript. All authors were responsible for the conception, design of the study, performed statistical analysis, contributed to the interpretation of findings, made critical revisions, and have read and agreed to the published version of the manuscript.
